# Do health insurances reduce catastrophic health expenditure in China? A systematic evidence synthesis

**DOI:** 10.1371/journal.pone.0239461

**Published:** 2020-09-24

**Authors:** Furong Li, Yuxuan Wu, Qingqing Yuan, Kun Zou, Min Yang, Dandi Chen

**Affiliations:** 1 Department of Health Policy and Management, West China school of Public Health and West China Fourth Hospital, Sichuan University, Chengdu, Sichuan, China; 2 West China Hospital, Sichuan University, Chengdu, Sichuan, China; 3 West China Research Centre for Rural Health Development, Sichuan University, Chengdu, Sichuan, China; 4 Faculty of Health, Art and Design, Swinbune Technology University, Melbourne, Australia; Wuhan University, CHINA

## Abstract

**Objective:**

To examine the association of health insurances on catastrophic health expenditure (CHE), and compares that among different health insurances in the last two decades in China.

**Methods:**

The systematic review was conducted according to the Cochrane Handbook and reported according to PRISMA. We searched English and Chinese literature databases including PubMed, EM base, web of science, CNKI, Wan fang, VIP and CBM (Sino Med) for empirical studies on the association between health insurance and CHE from January 2000 to June 2020. Study selection, data extraction and quality appraisal were conducted by two reviewers. The secular trend of CHE rate and comparisons between population with different health insurances were conducted using meta-analysis, subgroup analysis and meta-regression.

**Results:**

A total of 4874 citations were obtained, and finally 30 eligible studies with 633917 participants were included. The overall CHE rate was 13.6% (95% CI: 13.1% - 14.0%) from Jan 2000 to June 2020, 12.8% (95% CI: 12.2% - 13.3%) for people with health insurance compared with 16.2% (95% CI:15.4% - 16.9%) for people without health insurance. For types of insurance, the CHE rate was 13.0% (95% CI: 12.4% - 13.6%) for people with new rural cooperative medical scheme (NCMS), 11.9% (95% CI: 9.3% - 14.5%) for urban employees health insurance (UEBMI), 12.0% (95% CI: 8.3% - 15.6%) for urban residents health insurance (URBMI), and 18.0% (95% CI: - 4.5% - 31.5%) for commercial insurance. However, the CHE rate in China has increased in the past 20 years, even adjusted for other factors. The CHE rate of people with NCMS has increased significantly more than people with UEBMI and URBMI.

**Conclusion:**

In the past 20 years, the basic health insurance plan has reduce the rate of CHE to a certain extent, but due to the rapid increase in medical costs and the release of health needs in recent years, it masks the role of health insurance. More efforts are needed to control unreasonable medical demand and rising costs.

## Introduction

Every year, about 150 million people in the world spend heavily on health care, and 100 million people are pushed below the poverty line [1]. With rapid population aging in China, the health demands and healthcare expenditure has raising rapidly. However, "it is difficult and expensive to seek medical adviser", has become public concern in the Chinese society [2]. Research found that in 2003, 2008 and 2013, 13.6%,15.1% and 13.8% of households had catastrophic health expenditure (CHE) in China, respectively, of which the poverty rate due to illness was 8.6%. The households with CHE spent an average of 64% of their non-survival expenditure on medical expenses, suggesting the severity of this problem [3].

In order to solve this problem, the Chinese government has made unremitting efforts and made a series of policies involving three basic health insurance schemes, increasing the proportion of reimbursement, making national essential drug lists system, and expanding reimbursement scope of health insurance of new treatments to improve the level of financial protection.

In 1998, the State Council started comprehensive reform of basic health insurances for urban employees (UEBMI) [4]. By 2019, 329.25 million urban workers were involved in the insurance scheme. In rural population, the new rural cooperative medical scheme (NCMS) was piloted in 2002, which was expanded to national wide in 2008, and by 2014, more than 98.9% of rural population was involved (Data from the National Bureau of Statistics). In cities, basic health insurance for urban residents (URBMI) was piloted in 2007. In 2019, 102.51 million urban residents including non-employed or self-employed residents, college students, and children participated in URBMI. Since then, China's basic health insurance system has achieved universal coverage of the population [5].

However, there are some differences among the three basic health insurance systems. First, the financing mechanism is different. UEBMI is jointly paid by employers and individuals. The employers' payment amount is about 6% of employees' wages, while individuals' payment amount is about 2% of employees' wages. In terms of financial level, UEBMI is the highest (2840 yuan in average in 2014), URBMI is in the middle (524 yuan in average in 2014), and NCMS is the lowest (410 yuan in average in 2014). Higher financing level corresponds to higher payment guarantee ability. Second, the compensation level is different. Among them, the out-of-pocket (OOP) payment of UEBMI is the lowest (average 15% in 2015), which compensation ability is the strongest. However, the compensation ability of NCMS (OOP 25% in average in 2015), and URBMI (OOP 30% in average in 2015) was inferior to the former [6].

Third, the reimbursement mechanism is different. UEBMI and URBMI implement a direct settlement method combining personal account and co-ordination fund payment. The health insurance fund regularly pays the insured's personal account for use of outpatient or pharmacy services. The hospitalization expense is paid by the co-ordination fund. The insured only need to pay the OOP when leaving the hospital, and the rest is settled directly by the health insurance fund and the hospital. However, the NCMS adopts the mode that the insured pay in advance when leaving the hospital and being reimbursed after, and the reimbursement process is relatively complex [7]. Fourth, the way of participation is different. For URBMI and NCMS, voluntary participation is adopted, the government will give a certain share of subsidies to encourage more urban residents to participate (S1 Table). All these differences among the three basic insurance schemes may have impact on the ability of preventing CHE for corresponding population.

It has been ten years since the new health reform in China from 2009. Though empirical studies have been conducted to evaluate the effect of health insurance on CHE in China, systematic evaluation of the effect is lacking. Therefore, this study was conducted to systematically examine the effect of health insurance on CHE in China in the last two decades by synthesis of all relevant quantitative empirical evidence.

## Methods

A systematic review was conducted according to Cochrane Handbook of Systematic reviews [8] and reported according to the standard of preferred reporting items for systematic reviews and meta-analyses (PRISMA) [9].

### Inclusion and exclusion criteria

The inclusion criteria were: (1) participants were residents from mainland China; (2) studies reported the CHE rate and types of health insurance; (3) study design including: randomised controlled trial (RCT), non-RCT, controlled before-after study, cohort study, interrupted time series study and cross-sectional study. Languages were restricted to English and Chinses as they were the main publication languages of studies from mainland China. Duplicate publications were excluded.

### Literature search

We systematically searched Chinese literature databases of CNKI, Wan fang and VIP, and English literature databases including PubMed, EMbase, and Web of Science from January 2000 to June 2020. We used terms of "catastrophic health expenditure", "catastrophic medical expenditure", "impoverishing health expenditure", "poverty due to illness" and "returning to poverty due to illness" as keywords (see S2 Table). In addition, reference lists of included studies were scanned for more eligible studies (S2 Table)

### Study selection

Study selection was conducted by one reviewer (YXW) and checked by another (FRL or QQY). First, title and abstract of citations were scanned, obvious irrelevant studies were excluded. Then, full texts of potentially eligible studies were read and selected according to the inclusion and exclusion criteria. Different opinions were decided through discussion or consultation of a senior reviewer (KZ or DDC).

### Data extraction and quality evaluation

We used standardized Excel form to extract data from included studies including: study author, publication year, region, participants, sample size, income level, types of health insurance, definition of CHE, CHE rate, and odds ratio (OR). One reviewer (YXW) evaluated the quality of included studies using the AHRQ scale and the NOS scale, which was checked by a second reviewer (FRL or QQY). Different opinions of researchers were resolved by discussion. If there were multiple reports using the same data source, the most comprehensive publication was used in the analysis.

### Statistical analysis

The pooled CHE rate and 95% confidence interval (CI) was estimated using meta-analysis. Heterogeneity of studies was examined using χ^2^ test and I^2^ (I^2^ >50% as significant heterogeneity). Random-effect model was used for meta-analysis when there was significant heterogeneity, otherwise fixed-effect model was used.

First, the total CHE rate in China from 2000 to 2020 was estimated. Then, the secular trend of CHE rate was explored. Subgroup analyses were also conducted to investigate the difference of CHE rate between insured and non-insured, and by types of health insurance. Further explore were made on the secular trend of CHE rate among population with different health insurance status. We also performed meta-regression analysis to explore the sources of heterogeneity. The definition of CHE in the study, the year of data collection, whether the study population had special type of disease, whether the study participants were elderly, and types of insurance were included in the meta-regression analysis. Sensitivity analysis was conducted by eliminating the study one by one to examine the influence of each study on the stability of the overall rate. Publication bias was examined using Egger′s test. All analyses were performed using STATA15.0.

## Result

### Study selection

**Fig 1:**
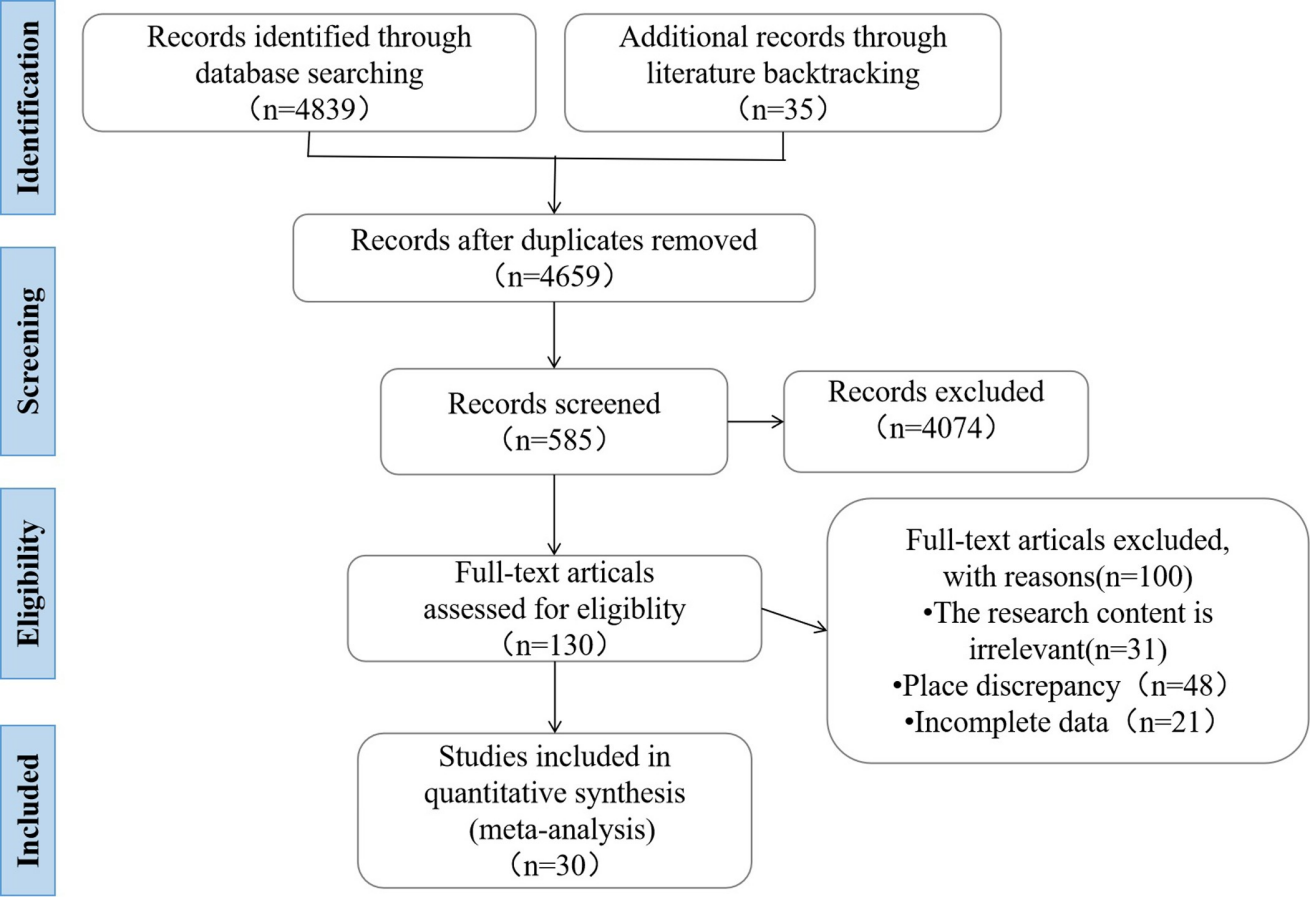
Flow chart of study selection.

In total, 4874 citations were obtained through systematic literature search, among which 585 citations were included after preliminary screening of titles and abstracts. Finally, 30 studies were included after reading the full texts (Fig 1).

### Characteristics of includes studies

The publication year was from 2000 to June 2020, including 22 studies for residents with NCMS, 9 studies for UEBMI, 9 studies for URBMI, 1 study for commercial health insurance, and 30 for non-insured. Six studies were conducted nationwide, 3 were cross-provinces including Hunan, Shandong and Ningxia, and 20 in individual province or metropolises including Shanghai, Shijiazhuang, Beijing, Hangzhou or others. For participants, 12 studies included general population, 2 involved rural population or insurers of NCMS and 13 included hospital patients of major diseases.

Among all, 9 studies used the WHO definition of CHE as ‘The proportion of family's medical and health expenses paid in cash to family's consumption expenditure is more than 40%’ [9], 4 studies defined CHE as ‘The total expenditure of family health accounts for more than 40% of the family's affordability’ and 12 studies defined CHE as ‘The proportion of self-paid medical and health expenses in the non-food consumption expenditure is more than 40%’. After discussion, we considered that 15 studies may overestimate the rate of CHE compared to the WHO definition of CHE, and one study may underestimate it [10].

For cross-sectional study, there were 6, 6 and 7 studies with NOS quality scores of 3, 4 and 5, respectively, and 9 studies with 6 (full score of 11). The quality score of the 1 cohort study was 5 (full score 9). Overall, the quality of the studies included was common (Table 1).

**Table 1:** Characteristics of included studies.

Author year	Region	Research type	Participants	N	Health insurance	Definition of CHE	Quality score
Fang 2004 [11]	China	Cross sectional	At least one member of the family participates in social insurance	9700	Any kind of health insurance	The total expenditure of family health accounts for more than 40% of the family's affordability	6
Jia 2006 [12]	Shanghai	Cross sectional	General population	3494	NCMS	The proportion of hospitalization expenses paid by family cash in household consumption expenditure is more than 40%	3
Sun 2007 [13]	Shandong	Cross sectional	Population in pilot counties of NCMS	375	NCMS	Household health expenditure exceeds 40.00% of capacity to pay	3
Sheng 2010 [14]	Huaihua	Cross sectional	General population	320	NCMS	Out of pocket medical expenses account for more than 40% of household non- food expenses	4
Chu 2010 [15]	Shandong, Ningxia	Cohort	General population	6137	NCMS	The proportion of the medical and health expenses paid by the family to the non-food expenses of the family is more than 40%	5
Wang 2012 [16]	Shijiazhuang	Cross sectional	Urban family	305	UEBMI, URBMI	Family OOP medical expenses exceed non-food expenses by 40%	5
Xie 2012 [17]	Chengkou County, Chongqing	Cross sectional	Administrative staff and inpatients of NCMS	125	NCMS	Health expenses account for more than 50% of family income	3
Yan 2012 [18]	Meixian County, Shanxi Province	Cross sectional	General population	2134	NCMS、UEBMI、URBMI	More than 40% of non-food expenses are paid by families in cash	4
Ye Li 2012 [19]	China	Cross sectional	General population	56400	NCMS、UEBMI、URBMI	Out of pocket medical expenses account for more than 40% of household non-food expenses	6
Guo 2013 [20]	Zhangqiu City, Changqing District, Pingyin County, Shandong Province	Cross sectional	Patients with serious illness	263	NCMS	Household cash health expenditure accounts for more than 40% of household non-food expenditure	4
Shi 2013 [21]	Yulong County, Yunnan Province	Cross sectional	General population	300	NCMS	The actual medical expenses paid by the family in the past year exceed 40% of the total expenditure of the family	4
Yan 2013 [22]	Meixian County, Shanxi Province	Cross sectional	General population	2134	NCMS	More than 40% of non-food expenses are paid by families in cash	4
Chen 2014 [23]	Zhejiang, Hubei, Chongqing	Cross sectional	General population	1661	NCMS	The proportion of self-paid medical and health expenses in the non-food consumption expenditure is more than 40%	5
Fan 2014 [24]	Hangzhou City	Case control	Over 60 years old	530	UEBMI、URBMI	Over 40% of the elderly's household non-food expenditure	6
Fu 2014 [25]	Heilongjiang Province	Cross sectional	Households with catastrophic expenditure in rural areas	296	Any kind of social insurance	Family expenditure represents family standard of living, and 40% is regarded as the standard of catastrophic health expenditure	3
Jing 2014 [26]	Sangzhi County, Mayang county and Lanshan County, Hunan Province	Cross sectional	General population	400	NCMS	The ratio of household health care expenditure to household consumption expenditure is more than 40%	6
Chen 2015 [27]	Anhui Province	Cross sectional	General population	518820	NCMS	RC = (I-S) × h, RC is the borderline of economic risk, I is the per capita disposable income, s is the minimum living insurance standard for farmers, and H is the per capita population	3
Luo 2016 [28]	Hubei province	Cross sectional	General population	441	NCMS	The proportion of health expenditure of patients with serious diseases in the family's ability to pay is more than 40%	4
Wang 2016 [29]	China	Cross sectional	Rural families	787	NCMS	Health spending accounts for more than 40% of household income	5
Wu 2016 [30]	Nanchang	Cross sectional	Lung cancer patients	203	Any kind of social insurance	Over 40% of the family's medical and health expenses are self-financing	6
Zhou 2016 [31]	Zigong City	Cross sectional	Rural families in Fushun County	2244	NCMS	Household cash health expenditure accounts for 40% of household non-food consumption expenditure	6
Gao 2017 [32]	China	Cross sectional	Patients with serious illness	497	NCMS	Gold health expenditure accounts for 40% of household consumption	3
Li 2017 [33]	Shanxi Province	Cross sectional	General population	55	NCMS、UEBMI、URBMI	Out of pocket medical expenses account for more than 40% of household non-food expenses	5
Li 2017 [34]	Jiangsu Province	Cross sectional	Patients hospitalized due to serious illness	400	Any kind of health insurance	The proportion of inpatients' out of pocket medical expenses in the family's annual non-food expenditure is more than 40%	2
Peng 2017 [35]	Zhongjiang County	Cross sectional	Inpatients in a tertiary hospital in Deyang City	1902	NCMS	Cash out of pocket expenses exceed the annual per capita net income of farmers	6
Wang 2018 [36]	Fushun County	Cross sectional	Rural families	999	NCMS	The proportion of family's medical and health expenses paid in cash to family's consumption expenditure is more than 40%	6
Wu 2018 [37]	Guangxi Zhuang Autonomous Region	Cross sectional	Inpatients with myocardial infarction	500	NCMS、UEBMI、URBMI、commercial insurance	The proportion of inpatients' out of pocket medical expenses in the family's annual non-food expenditure is more than 40%	5
Ding 2019 [7]	China	Cross sectional	Group of the elderly	2564	NCMS、UEBMI、URBMI	The proportion of medical and health expenses paid by family cash in family expenditure is more than 40%	5
Wang 2019 [38]	China	Cross sectional	Adult	10364	NCMS、UEBMI、URBMI	The proportion of medical and health expenses paid by family cash in family expenditure is more than 40%	5
Meiyan Ma 2020 [39]	China	Cross sectional	Middle aged and old people	9167	NCMS、UEBMI、URBMI	The proportion of medical and health expenses paid by family cash in family expenditure is more than 40%	6

NCMS: New rural cooperative medical scheme, UEBMI: Basic health insurance for urban employees, URBMI: Basic health insurance for urban residents, OOP: out-of-pocket payment.

### Rate of catastrophic health expenditure in the last two decades

**Fig 2:**
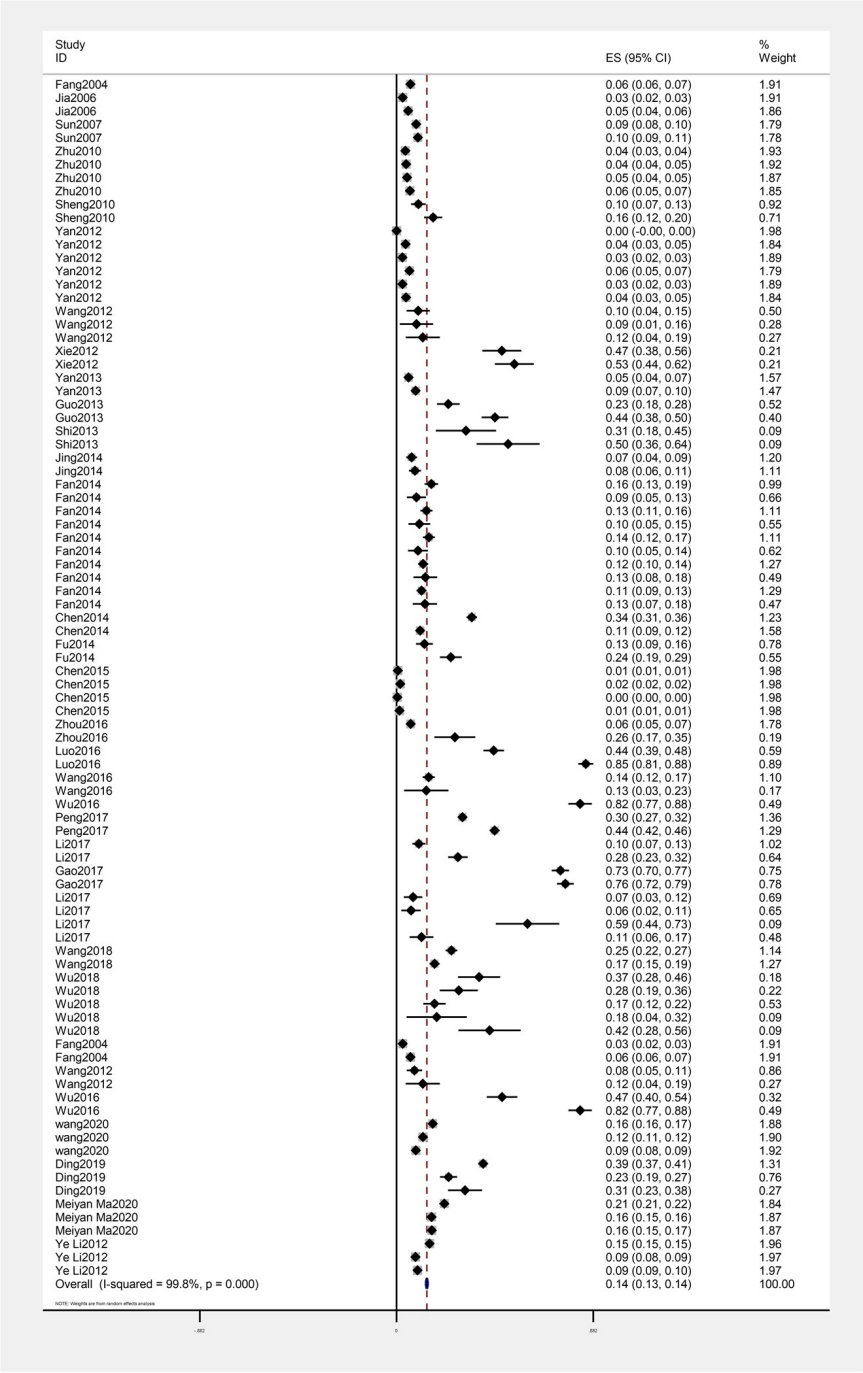
Rate of catastrophic health expenditure of Chinese residents from 2000–2020.

There was significant heterogeneity among included studies, thus random-effect model was used. The rate of CHE of Chinese residents was 13.6% (95% CI: 13.1% - 14.0%) from 2000 to 2020 (Fig 2).

### Catastrophic health expenditure rate and health insurance

**Fig 3:**
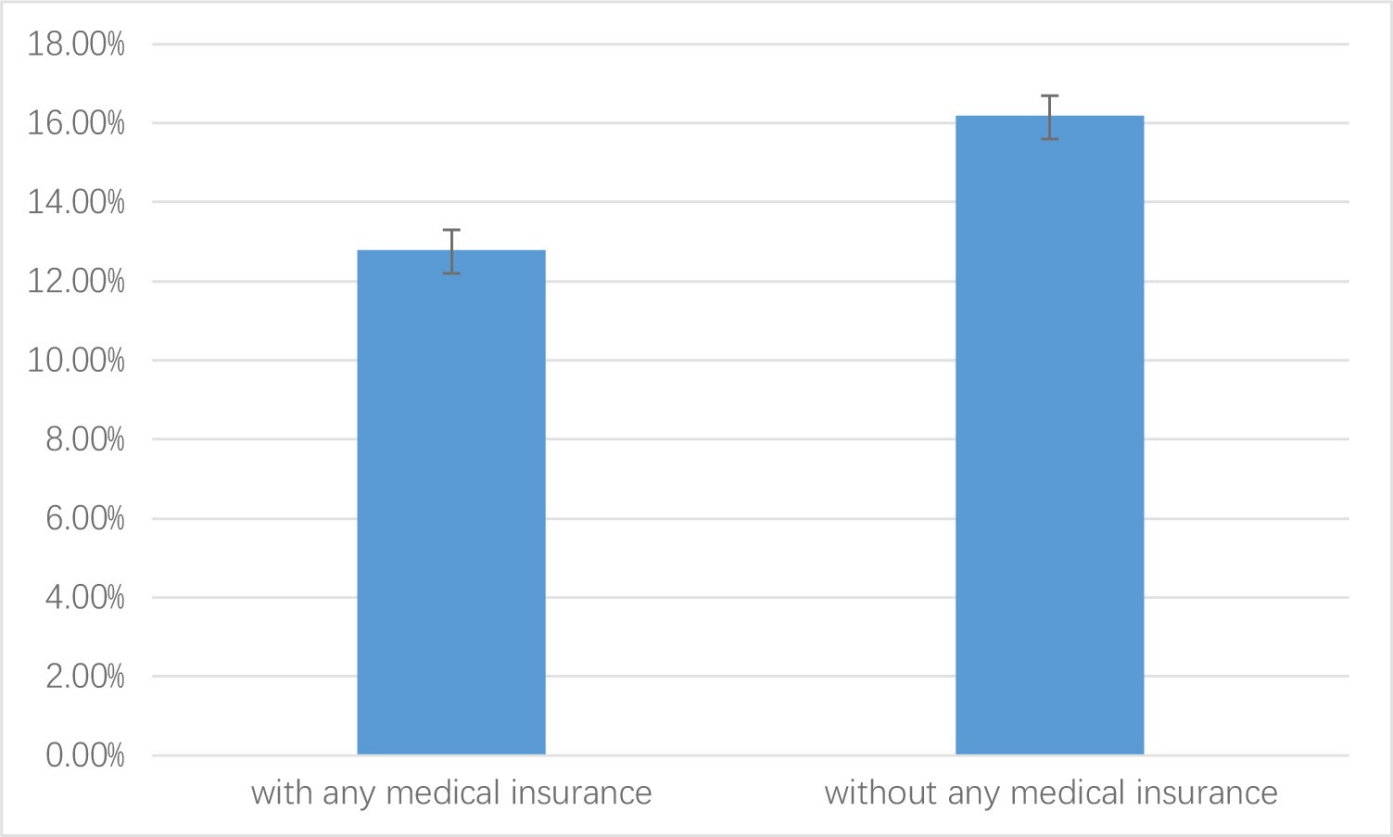
Health insurance and the rate of catastrophic health expenditure.

Subgroup analysis showed that the rate of CHE in people who with any health insurance (12.8% 95%CI: 12.2% - 13.3%) was significantly lower than who without (16.2%, 95%CI: 15.4% - 16.9%) (Fig 3). Therefore, health insurance can reduce the rate of CHE (S1 Fig).

### Catastrophic health expenditure by types of health insurance

**Fig 4:**
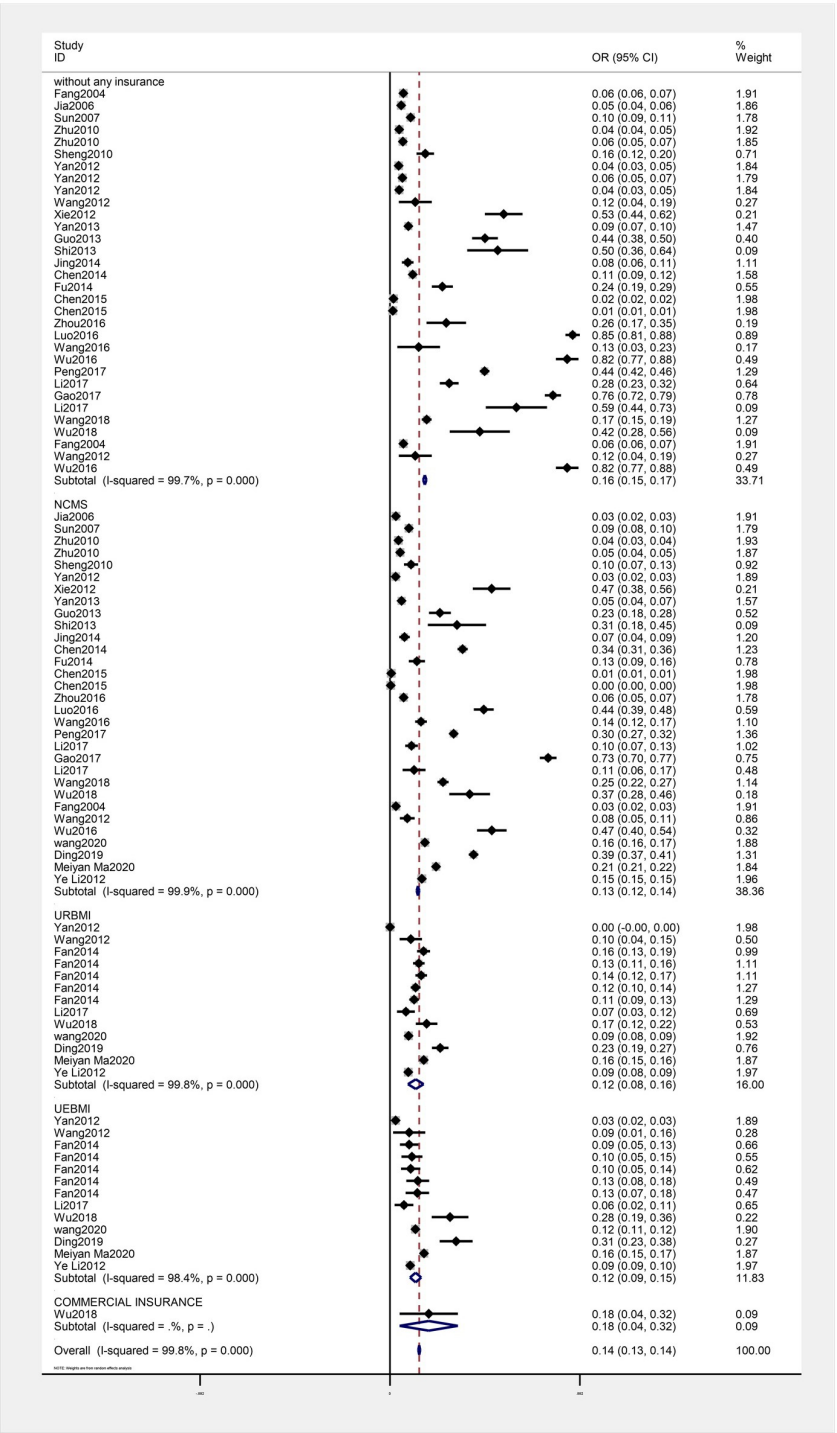
Rate of catastrophic health expenditure by types of health insurance.

Subgroup analysis showed that the CHE rate from the highest to the lowest was 18.0% (95CI: 4.5% - 31.5%) for commercial insurance,16.2% (15.4% - 16.9%) for people without any insurance, 13.0% (12.4% - 13.6%) for NCMS,12.0% (95% CI: 8.3% - 15.6%) for URBMI and 11.9% (9.3% - 14.5%) for UEBMI (Fig 4).

### Secular trend of rate of health catastrophic expenditure and health insurance

For the people who with health insurances, the rate of CHE was 3.2% (2.4%, 4.0%) from 2004 to 2006, 10.0% (7.5%, 12.5%) from 2007 to 2009, 10.1% (7.8%, 12.4%) from 2010 to 2012, 14.0% (13.4%, 14.6%) from 2013 to 2016, and 23.9% (12.6%, 35.2%) from 2017 to 2020. For people without health insurance, the rate of CHE was 5.5% (4.5%, 6.5%) from 2004 to 2006, 7.8% (4.3%, 11.3%) from 2007 to 2009, 11.2% (8.2%, 14.3%) from 2010 to 2012, 19.2% (18.1%, 20.3%) from 2013 to 2016, 43.9% (25.0%, 62.8%) from 2017 to 2020.

**Fig 5:**
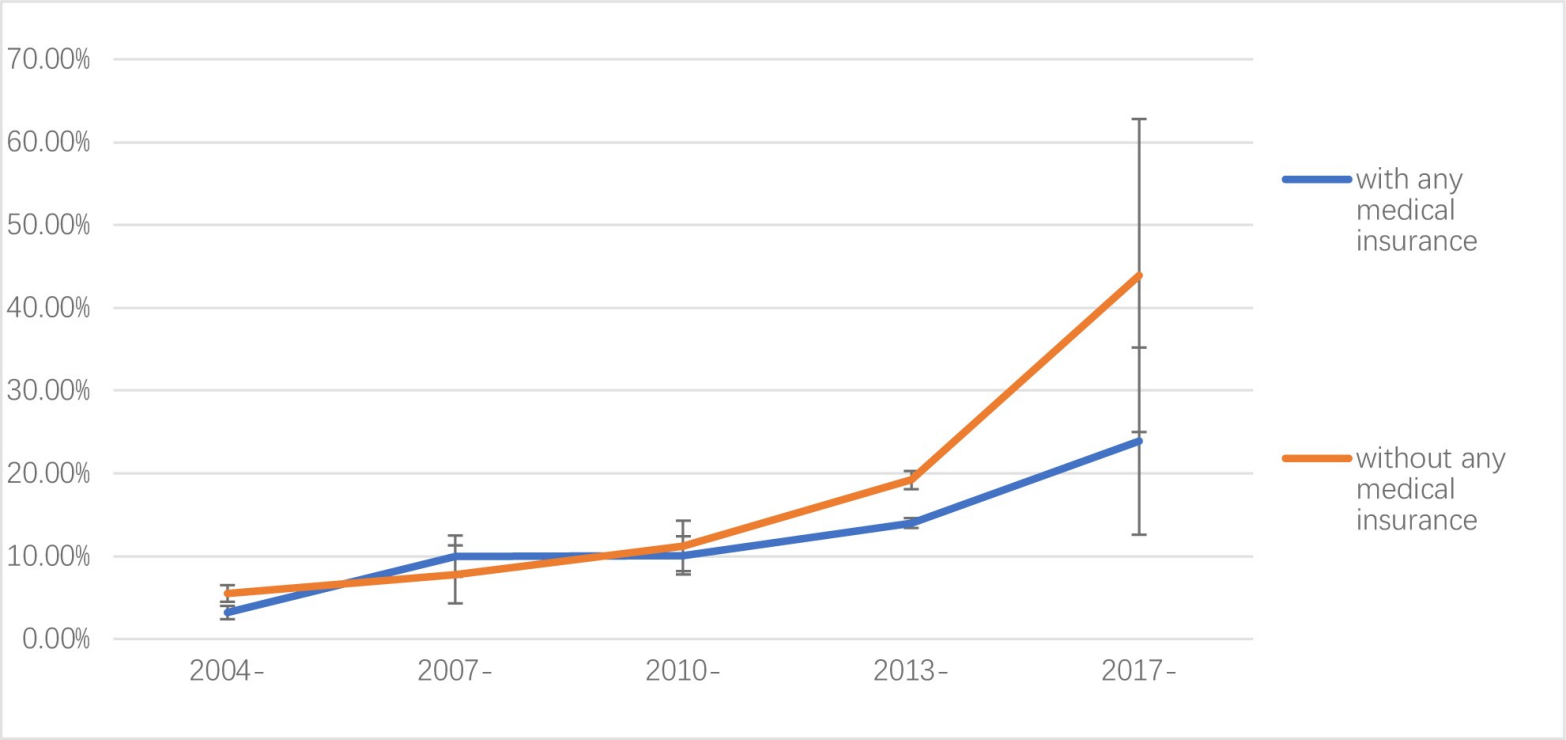
Secular trend of rate of catastrophic health expenditure and health insurance.

Since 2010, the rate of CHE in the population with health insurance was significantly lower than those without. Although both of them were on the rise, the rate of CHE in the population without health insurance was increasing faster and the gap between the two became wider (Fig 5).

### Secular trend of catastrophic health expenditure by type of insurance

**Fig 6:**
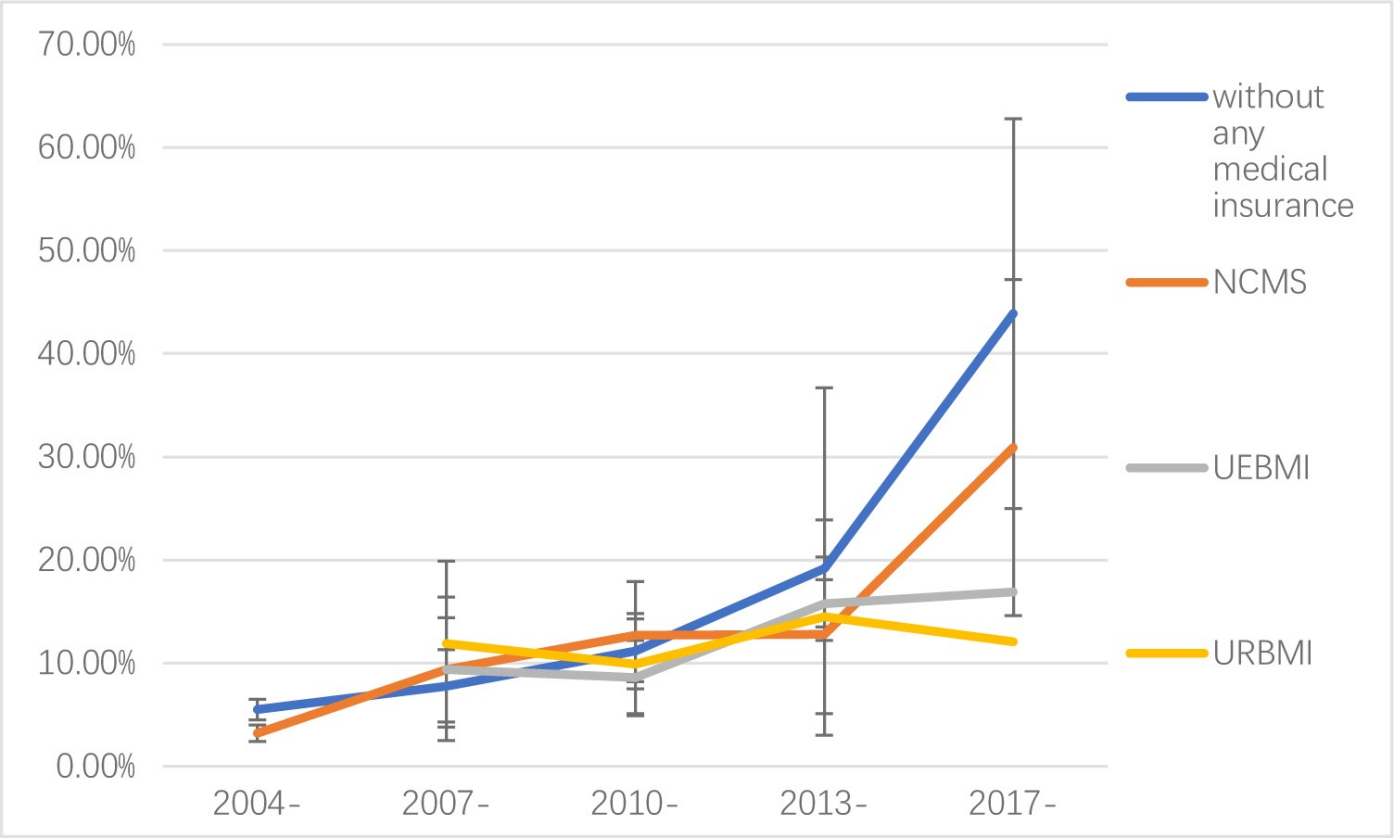
Secular trend of rate of catastrophic health expenditure by types of health insurance.

The results showed that the rate of CHE of all subgroups increased with time. The CHE rate of people who did not participate in the health insurance increased more and faster, especially after 2013, followed by NCMS, UEBMI and URBMI. Due to lack of data, the trend of CHE rate of commercial health insurance could not be obtained (Fig 6).

### Meta-regression of factors associated with rate of catastrophic health expenditure

There was a positive association between year (2007-: beta = 3.68, 95%CI:1.44~9.39, p = 0.007. 2010-: beta = 3.04, 95% CI: 1.34~6.92, p = 0.009. 2013-: beta = 3.34, 95% CI: 1.55~7.17, p = 0.002. 2017-: beta = 5.52, 95% CI: 2.26~13.48, p<0.001), disease (beta = 2.53, 95% CI: 1.35~4.74, p = 0.004), age (beta = 2.62,95%CI:1.36~5.05, p = 0.005), definition CHE (CHE (higher): beta = 3.54, 95% CI: 1.01~12.41, p = 0.048. CHE (lower): beta = 2.09, 95% CI: 1.34~3.25, p = 0.001) and the rate of CHE, while there was a negative but non-significant association between health insurance (P>0.05, except for commercial health insurance p = 0.028) and the rate of CHE. It is inferred that the heterogeneity between studies is related to the age, diseases, different definitions of CHE and the year, which could explain 43.37% of the heterogeneity between the study results (Table 2).

**Table 2:** Meta-regression of factors associated with rate of catastrophic health expenditure.

Variable	Coefficient	P	[95% Conf. Interval]	
CHE (lower threshold)	1.26	0.048	0.01	2.52
CHE (higher threshold)	0.74	0.001	0.30	1.18
Elderly	0.96	0.005	0.31	1.62
Special disease group	0.93	0.004	0.30	1.56
2007-	1.30	0.007	0.36	2.24
2010-	1.11	0.009	0.29	1.93
2013-	1.21	0.002	0.44	1.97
2017-	1.71	<0.001	0.82	2.60
NCMS	-0.70	0.478	-2.65	1.25
UEBMI	-0.37	0.105	-0.82	0.08
URBMI	-0.58	0.103	-1.28	0.12
Commercial insurance	-0.80	0.028	-1.51	-0.09

CHE: catastrophic health expenditure, NCMS: New rural cooperative medical scheme UEBMI: Basic health insurance for urban employees, URBMI: Basic health insurance for urban residents.

We further explored the relationship between the growth rate of China's per capita health expenditure and per capita disposable income in recent years, and found that in recent years, China’s per capita health expenditure and per capita disposable income have increased to varying degrees, and the growth rate of per capita health expenditure was much higher than that of per capita disposable income. It shown that the excessive growth of health expenses may be an important reason for the occurrence of CHE (S2 Fig).

### Publication bias

Egger’s test indicated that the studies included had a significant publication bias. (t = 2.62, P = 0.01) (S3 Fig).

## Discussion

There were five main findings of this study. First, health insurances provided financial protection against CHE in China in the last two decades. Second, the financial protective effect against CHE was strongest in UEBMI, followed by URBMI, and NCMS. Third, the rate of CHE for Chinese residents has gradually increased, but the growth rate of people who with health insurance was slower than who without. Fourth, the gap between NCMS and the other two types of health insurances to prevent CHE was increasing. Fifth, major illness and old age were associated with increases risk of CHE.

The rate of CHE is significantly different between those who participate in health insurance and those who do not. However, the rate of CHE has been increasing still, whether participating in or not in health insurances. This was consistent with the finding of Wang and Xu, who reported that basic health insurance schemes did not reduce the risk of CHE and may even increase it using China Health and Retirement Longitudinal Study data [40, 41]. It may because the protective effect of health insurances has been offset by the rapid rise in medical expenditure, caused by developing economics [42], population aging [43, 44], increases healthcare needs, the use of new drugs and new materials[45], induced excessive medical services[46, 47] which runs counter to the original intention of health insurance [48], service utilization and rising price of medicine, devices and medical technologies [4, 49], and lack of health expenditure control measures [45]. The increase in medical expenditure includes reasonable and unreasonable ones.

We also found that the growth rate of per capita health expenditure was much higher than the growth rate of per capita disposable income in the last decades, under this circumstance, any medical insurance plan will face a huge risk of insolvency [50].

Another important reason is that the scope of medical security in my country is currently limited. Outpatient services and some medicines are not included in the reimbursement scope. Because of the limited, households having a chronically ill member may refrain from seeking care until advanced illness sets in. Extending insurance coverage to long-term care for chronically ill patients, outpatient services, routine essential drugs and rehabilitation services should be a priority [19].

For secular trend, the rate of CHE for all three types of health insurances were on the rise, for which NCMS was the most obvious, indicating that the protective effect of NCMS on CHE is the least. The main reason may due to the inconsistency in the system design of the three insurance schemes [51]. The first reason is the compensation methods of NCMS in China [24]. Primary medical institutions are incapable of treating diseases that cause CHE, and must go to high-level medical institutions, but the reimbursement ratio of high-level medical institutions is lower than that of primary medical institutions [52]. Therefore, the risk of CHE is increased.

Second, NCMS insured are rural residents and there has been inequity of access to health services in rural China compared with urban residents. The lack of medical resources and people's poor awareness of disease prevention and control may delay the diagnosis of diseases until very severe or advanced stage, which required higher medical costs than early detected ones [53].

We also found that patients suffering from major diseases and old age were associated with higher rate of CHE. This is consistent with the research finding from Yu Zhen and colleagues in Zhao Tong City, Yunnan Province [54]. Because the elderly was more likely to suffer from chronic diseases, long-term medication and treatment may lead to higher medical expenses.

In order to control and reduce the rate of CHE, policies and measures must be made to control the unreasonable increase of total medical expenses and increase the reimbursement of high-cost diseases. Secondly, measures are needed to unified health insurance systems for serious illnesses, so that severely ill patients may get more economic protection and reduce the risk of CHE.

### Strengths and limitations

This study had several strengths. First, as our best knowledge, this was the first systematical review examining the association of health insurance and CHE in China. Second, this study was performed complying to international recognized methodological guideline and standards which provided by far the most comprehensive evidence of health insurance and CHE in China. However, there were several limitations. First, there was large heterogeneity between study included, which could not be solely explained by varied study characteristics. Second, the number of studies for some of the subgroups was small, and with high risk of publication bias, which may affect the reliability of the results.

## Conclusion

In the past 20 years, the Chinese government has adopted a series of measures to reduce the rate of CHE. The coverage and reimbursement ratio of health insurance have been improved and show protective role against CHE compared with no insurance, but the rate of CHE have not reduced significantly. This is mainly caused by reasonable and unreasonable increase in medical costs, increased disease burden and limited reimbursement scope. Health policies and measures are warranted to control unreasonable medical costs and further improve the health insurance reimbursement system. In terms of research, more research on the impact of the levels of financial protection on CHE, and the impact of integration of different types of health insurance on CHE is needed.

## Supporting information

S1 Fig Impact of participation in health insurance on the prevalence of catastrophic health expenditure. (DOCX)

S2 Fig Per capita disposable income and per capita health expenditure growth. (DOCX)

S3 Fig Funnel plot of publication bias. (DOCX)

S1 Table Characteristics of basic health insurances schemes in China. (DOCX)

S2 Table Search strategy in PubMed. (DOCX)

S3 Table Variable assignment and definition in meta regression analysis. (DOCX)

S1 Checklist (DOCX)
