# Supplementary material for: Do health insurances reduce catastrophic health expenditure in China? A systematic evidence synthesis
Source: PLoS One. 2020 Sep 24;15(9):e0239461. doi: 10.1371/journal.pone.0239461 (PMC7514005; doi:10.1371/journal.pone.0239461)
Supplement: S1 Fig — (DOCX) [file pone.0239461.s001.docx]

S1 Fig. Impact of participation in health insurance on the prevalence of catastrophic health expenditure
