# Supplementary material for: Do health insurances reduce catastrophic health expenditure in China? A systematic evidence synthesis
Source: PLoS One. 2020 Sep 24;15(9):e0239461. doi: 10.1371/journal.pone.0239461 (PMC7514005; doi:10.1371/journal.pone.0239461)
Supplement: S1 Table — (DOCX) [file pone.0239461.s004.docx]

S1 Table. Characteristics of basic health insurances schemes in China

|  | New rural cooperative medical scheme | Basic health insurances for urban employees | Basic health insurance for urban residents |
| --- | --- | --- | --- |
| Participants | Rural residents | Urban employees | non-employed or self-employed residents in urban areas |
| Year of initiative (pilot) | 2002 | 1998 | 2007 |
| Year of nation-wide implementation | 2008 | 1999 | 2010 |
| Population coverage by year 2019 | 736 million in 2014 | 329.26 million | 1025.1 million |
| Health financing methods | personal contributions (22%） and financial subsidies (78%）in 2004 | Individual (about 6% of employees' wages) and employer payment (about 2% of employees' wages) | Personal contributions (24%) and financial subsidies (76 %) in 2015 |
| Government subsidies | Yes（78%） | No | Yes （76%） |
| Participation requirements | Voluntary | Compulsory | Voluntary |
| Reimbursement ratio | 75% for hospitalization expense in 2015 | In 2019, Within the scope of the employee medical insurance policy, the hospitalization expense fund pays 85.8%, the actual hospitalization expense fund pays 75.6%, and the personal burden is 24.4%. The payment of hospitalization expense fund within the policy scope of the second-level and below-level medical institutions was 87.2% and 89.3% respectively, which was 2.2 and 4.3 percentage points higher than that of the third-level medical institutions. | 70% for hospitalization expense in 2015 |
| Per capita funding level | 410 yuan per person per year in 2014 | About 8% of employees' wages | 500 yuan per person per year in 2015 |

Notes：The data comes from the National Bureau of Statistics, the National Medical Security Bureau, the Ministry of Human Resources and Social Security of the People's Republic of China.
