# Supplementary material for: Do health insurances reduce catastrophic health expenditure in China? A systematic evidence synthesis
Source: PLoS One. 2020 Sep 24;15(9):e0239461. doi: 10.1371/journal.pone.0239461 (PMC7514005; doi:10.1371/journal.pone.0239461)
Supplement: S2 Table — (DOCX) [file pone.0239461.s005.docx]

S2 Table．Search strategy in PubMed

| Keywords |
| --- |
| 1、Catastrophic health expenditure |
| 2、Catastrophic medical expenses |
| 3、Poverty-causing health expenditure |
| 4、Poverty due to illness |
| 5、Return to poverty due to illness |
| 6、1 or 2 or 3 or 4 or 5 |
| 7、China |
| 8、6 and 7 |
