# Supplementary material for: Do health insurances reduce catastrophic health expenditure in China? A systematic evidence synthesis
Source: PLoS One. 2020 Sep 24;15(9):e0239461. doi: 10.1371/journal.pone.0239461 (PMC7514005; doi:10.1371/journal.pone.0239461)
Supplement: S3 Table — (DOCX) [file pone.0239461.s006.docx]

S3 Table．Variable assignment and definition in Meta regression analysis

| Variable | Assignment | Definition |
| --- | --- | --- |
| CHE(WHO) | 0 | This study proposed the definition of CHE by WHO |

| CHE (lower threshold) | 1 | According to the research, this study's definition of CHE may have underestimated the prevalence of CHE compared with the WHO definition (40%) |
| --- | --- | --- |

| CHE (higher threshold) | 2 | According to the research, this study's definition of CHE may have overestimated the prevalence of CHE compared with the WHO definition (40%) |
| --- | --- | --- |

| General people | 0 | The object of this study is the general population |
| --- | --- | --- |

| Elderly | 1 | The research object of this study is the elderly over 60 years old |
| --- | --- | --- |

| Healthy people | 0 | The object of this study is not suffering from disease |
| --- | --- | --- |

| Special disease group | 1 | The research object of this study is patients with certain major diseases |
| --- | --- | --- |

| 2004- | 0 | The data for this study was between 2004 and 2006 |
| --- | --- | --- |

| 2007- | 1 | The data for this study was between 2007 and 2009 |
| --- | --- | --- |
| 2010- | 2 | The data for this study was between 2010 and 2012 |
| 2013- | 3 | The data for this study was between 2013 and 2016 |
| 2017- | 4 | The data for this study was between 2017 and 2020 |

| The uninsured | 0 | The object of this study is the uninsured population |
| --- | --- | --- |

| NCMS | 1 | The research object of this study is the NCMS insured population |
| --- | --- | --- |
| UEBMI | 2 | The research object of this study is the UEBMI insured population |
| URBMI | 3 | The research object of this study is the URBMI insured population |
| Commercial insurance | 4 | The research object of this study is the commercial insurance insured population |
